# Factors associated with secondhand smoke exposure among non-smoking employees in the workplace: A cross-sectional study in Qingdao, China

**DOI:** 10.1371/journal.pone.0263801

**Published:** 2022-08-25

**Authors:** Xiaocen Jia, Rui Wang, Xiaofei Qiu, Yiqing Huang, Yani Wang, Xiaorong Jia, Shanpeng Li, Yibo Wu, Fei Qi

**Affiliations:** 1 School of Public Health, Qingdao University, Qingdao, Shandong Province, China; 2 Qingdao Municipal Center for Disease Control and Prevention, Qingdao, Shandong Province, China; 3 School of Public Health, Peking University, Beijing, China; AIIMS: All India Institute of Medical Sciences, INDIA

## Abstract

**Objective:**

This study was conducted to describe secondhand smoke (SHS) exposure among non-smoking employees in the workplace, and identify factors related to SHS exposure in Qingdao.

**Methods:**

The study participants covered key non-smoking places stipulated in the “Qingdao City Smoking Control Regulations,” which included three categories: restaurants, bars, and office buildings. Airborne nicotine concentration in the workplace and saliva cotinine concentration of employees were measured. The questionnaire included employees’ demographic factors, smoke-free measures in the workplace, employers’ tobacco hazard knowledge, and attitudes towards smoke-free policy.

**Results:**

A total of 222 non-smoking employees and 46 non-smoking employers were included in the study. The median concentrations of airborne nicotine and salivary cotinine were 0.389 μg/m^3^ and 0.575 ng/mL, respectively. Educational status, average number of workplace smokers per day, exposure time to SHS in the workplace, and whether smoking and non-smoking areas were divided significantly related to airborne nicotine concentration. Age, educational status, exposure time to SHS in the workplace, tobacco control training and publicity, and whether the employers support the “Qingdao Tobacco Control Regulation” were significantly related to salivary cotinine concentration.

**Conclusions:**

Despite the implementation of the “Qingdao Smoking Control Regulations” in 2013, the workplace remains an important location for SHS exposure. Interventions such as raising workers’ awareness of the risks associated with SHS exposure through health education and developing smoking prevention and cessation programs to reduce SHS exposure in the workplace are urgently needed.

## Introduction

Secondhand smoke (SHS), also referred to as environmental tobacco smoke, is a combination of smoke exhaled by smokers and smoke from the burning tips of cigarettes [1]. SHS is a serious health hazard, and can cause and/or worsen various health complications, including respiratory and cardiovascular diseases [2]. There is no risk-free level of SHS exposure, with even a brief exposure complications harmful to health [3]. Comprehensive smoke-free policies have been successful in protecting those who do not smoke in the workplace, and are the only way to fully protect their health [3]. The World Health Organization (WHO) therefore recommends the adoption and implementation of comprehensive national smoke-free legislation to protect people from SHS.

An increasing number of countries have enacted partial or comprehensive national smoke-free laws, which generally prohibit smoking in indoor public and workplaces, public transportation, and other public places, as stipulated by the WHO Framework Convention on Tobacco Control [4]. However, 50.9% of adults working indoors (216.9 million) were exposed to SHS in the workplace, according to the 2018 Global Adult Tobacco Survey. A study from the United States showed that 8.6% of non-smoking workers reported exposure to SHS frequently in the workplace, even in states with smoke-free laws at worksites [5]. The workplace remains the source of most SHS exposure for non-smoking adults [6], and exposure to SHS in the workplace has been recognized as one of the major occupational hazards contributing to the prevalence of occupational cancer among non-smokers [7]. Moreover, the workplace is one of the settings where many deaths related to SHS exposure have been reported. The International Labor Organization estimates that approximately 14% (approximately 200,000) of all work-related deaths due to diseases are related to SHS exposure in the workplace, worldwide [8].

China has the largest number of smokers worldwide [2], and it is estimated that 70% of Chinese adults are frequently exposed to SHS [9]. In recent years, local tobacco control policy initiatives have emerged in China. All Tier 1 cities, namely Beijing, Shanghai, and Shenzhen, have implemented local smoke-free policies. Qingdao, a new first-tier city (tier immediately below Tier 1) in eastern Shandong Province, enacted the smoke-free law on 31 August 2013, which prohibited smoking in indoor workplaces. The results of the 2014 Qingdao Adult Tobacco Epidemic Survey showed that the overall exposure rate to SHS in indoor workplaces in the early stage of Qingdao’s smoke-free law enactment was 32.7%. The workplace was observed as a strategic place for SHS exposure, as most adults spend more than half a day at their workplace [10]. In the past, self-reporting was typically used to assess employee exposure to SHS in the workplace, but self-reporting may under- or overestimate the actual exposure. This may be due to a lack of knowledge about how SHS is distributed in the workplace or inaccurate employee reports, indicating that objective measures are more reliable [11]. Air nicotine concentration can be measured to assess SHS exposure in a specific environment [12]. Nicotine is specific to tobacco smoke and often used to evaluate SHS in different indoor settings [13–15]. The biomarker cotinine, a nicotine metabolite, can be used to determine individual SHS exposure. The half-life of cotinine is approximately 20 h and is used as a biological index for the assessment of the SHS exposure rate [16].

To date, only a few studies have focused on the determinants of SHS exposure, even though this information is required to develop adequate public health policies to protect non-smokers. Studies on the determinants of SHS exposure in China are scarce. We therefore aimed was to describe SHS exposure among non-smoking employees in the workplace. SHS exposure of each non-smoking employee was assessed using a seven-day nicotine accumulation measurement and a measure of salivary cotinine. Our secondary aim was to explore the relationship between airborne nicotine concentrations in the workplace and employees’ salivary cotinine levels with reported employee demographic factors, smoke-free measures in the workplace, and employers’ tobacco hazard knowledge and attitudes towards smoke-free policies. The information provided by this study will allow for the development and implementation of targeted preventive measures to reduce workplace SHS exposure.

## Methods

### Study design

This survey was conducted in August 2020, Qingdao, China. The study participants covered the key non-smoking places stipulated in the “Qingdao City Smoking Control Regulations”. Convenience sampling methods were used to select 46 workplaces across three categories: restaurants, bars, and office buildings. The inclusion criteria were as follows: informed consent, no smoking (never smoked or quit smoking for more than half a year), age over 18 years, working time in the workplace for three hours or more on the test day, and exposure time to SHS in non-workplaces (such as homes and other public places) less than one hour on the test day. Those who were unwilling to participate in this research or unwilling to cooperate and those who were unable to communicate normally, such as those with text dyslexia, were excluded. Employers and employees completed a questionnaire to determine their demographic factors, smoking ban measures in the workplace, and knowledge of tobacco hazards. Airborne nicotine concentration in the workplace and saliva cotinine concentration of employees was measured. The survey instruments, protocols, and process for obtaining informed consent from participants were carried out in accordance with relevant guidelines and regulations and were approved by the Institutional Review Board of Qingdao Municipal Center for Disease Control and Prevention(CDC).

### Airborne nicotine sampling

Airborne nicotine concentrations were assessed using a passive sampling device that contained a 37 mm diameter filter treated with sodium bisulfate. The sampler was hung at 1.5 to 2 m from the ground and avoided places with no air circulation. After a seven-day sampling period, nicotine was enriched on the absorption membrane, and the absorption membrane samples were analyzed in an analytical laboratory. The laboratory is certified by the Johns Hopkins University Global Tobacco Control Institute for testing nicotine content in air samples. The total amount of nicotine absorbed by each filter was quantified using gas chromatography combined with mass spectrometry. Nicotine concentrations were calculated by dividing the total amount of nicotine by the rate of airflow and the length of time (in minutes) for which the device was installed [14]. This analysis procedure is certified by the ISO-17025, and has a nicotine limit of detection (LOD) of 0.02 μg/m^3^ for 1 week of exposure.

### Saliva sample collection

Before sample collection, the researchers wiped their hands thoroughly with baby wipes to minimize the chance of contamination. Saliva samples were collected using a professional saliva collection tube. The tested participants chewed the cotton swab for 45 s; thereafter the chewed cotton swab was placed back into the saliva collection tube, covered, and centrifuged to collect the saliva. The saliva samples were transported to the CDC on the same day for cryopreservation at -20°C and sent to the laboratory for cotinine concentration detection within 7 days of collection. The lower limit of salivary cotinine concentration was set at 0.1 ng/mL. Cotinine concentrations below the limit of quantification were designated half the level of quantification (0.05 ng/mL).

### Study variables

This study is exploratory in nature. In the multiple linear regression model, the dependent variables were airborne nicotine concentration in the workplace and salivary cotinine concentration among the employees. Information on the independent variables was assessed using a questionnaire survey. The first model aimed to assess demographic factors related to SHS exposure. The independent variables included information on sex (“male” and “female”), age groups (“18–30 years,” “31–45 years,” “46–60 years”), educational status (“low,” “middle” and “high”), average number of smokers per day in the workplace (“<1”, “1~10”, “>10”), exposure time of SHS in the workplace (“<1 h”, “1–6 h”, “>6 h”) and colleague smoking (“yes” and “no”). The second model aimed to assess smoke-free measures related to SHS exposure in the workplace. The independent variables included information on indoor smoking bans (“yes” and “no”), whether to divide smoking and non-smoking areas (“yes” and “no”), regulations on tobacco control (“yes” and “no”), leaders in charge of tobacco control (“yes” and “no”), tobacco control supervisor (“yes” and “no”), tobacco control training and publicity (“yes” and “no”), employees discourage smoking actively (“yes” and “no”). The third model aimed to assess the relationship between employers’ knowledge and attitudes towards smoking bans and SHS exposure in the workplace. The independent variables included information on awareness of whether to support the “Qingdao Tobacco Control Regulation”(“yes” and “no”), the effect of the “Qingdao Tobacco Control Regulation” on the workplace (“beneficial,” “unhelpful” and “no effect”), awareness of the “Qingdao Tobacco Control Regulation”(“yes” and “no”), awareness of the maximum fines of the “Qingdao Tobacco Control Regulations” (“yes” and “no”), and employers’ knowledge of tobacco hazard score.

### Data analysis

Statistical analyses were performed using SPSS 26.0. The significance level was set at p < 0.05. First, we checked the distribution of the airborne nicotine and salivary cotinine concentrations and found that they were non-normally distributed. Second, a logarithmic transformation of the skewed data was performed to approximate a normal distribution for statistical analyses. Correlations between airborne nicotine concentration and salivary cotinine concentrations were tested on the log-transformed data using Pearson correlation. Multiple linear regression analysis was used to determine whether any factor was significantly related to airborne nicotine and salivary cotinine concentrations. Participants with missing data were excluded only from the factor analysis associated with the missing variable.

## Results

### Participant demographics, airborne nicotine and salivary cotinine levels

Table 1 shows the demographics of all participants and the airborne nicotine and salivary cotinine levels within each demographic group. A total of 250 non-smoking employees and 46 employers from restaurants, bars, and office buildings participated in the study. Data related to salivary cotinine concentration was obtained from 235 employees. To control for misreported smoking status, we excluded 13 participants with saliva sample cotinine concentrations exceeding 15 ng/mL. In total, 222 employees were included in this study, of which, 32 had values below the detection limit (< 0.1 ng/mL), giving a value of 0.05 ng/mL for analysis. The median concentrations of airborne nicotine and salivary cotinine were 0.389 μg/m^3^ and 0.575 ng/ml, respectively, with a significant positive correlation of 0.382 (p < 0.01) between them.

**Table 1:** Summary of the demographic variables.

Demographic	Employee n (%)	Employer n (%)	Airborne nicotine concentration (μg/m^3^)	Salivary cotinine concentration (ng/mL)
**Sex**				
**Male**	78 (35.1)	33 (71.7)	0.381	0.649
**Female**	144 (64.9)	13 (28.3)	0.389	0.498
**Age**				
**18–30**	120 (54.1)	20 (43.5)	0.389	0.641
**31–45**	92 (41.4)	13 (28.3)	0.385	0.294
**46–60**	10 (4.5)	13 (28.3)	0.415	1.135
**Educational status**				
**Low**	27 (12.2)	4 (8.7)	0.623	1.048
**Middle**	75 (33.8)	16 (34.8)	0.623	0.839
**High**	120 (54.1)	26 (56.5)	0.306	0.317
**Type of workplace**				
**Restaurants**	88 (39.6)	21 (45.7)	0.914	1.165
**Bars**	44 (19.8)	10 (32.6)	0.166	0.395
**Office buildings**	90 (40.5)	15 (21.7)	0.328	0.210
**Average number of smokers per day in the workplace**				
**<1**	131 (59.0)		0.284	0.412
**1~10**	71 (32.0)		0.604	0.858
**>10**	20 (9.0)		0.905	0.864
**Exposure time of SHS in the workplace**				
**<1 h**	155 (65.8)		0.306	0.355
**1~6 h**	65 (27.9)		0.666	0.930
**>6 h**	15 (6.3)		1.055	1.311
**Colleague smoking**				
**Yes**	28 (12.6)		0.385	0.504
**No**	194 (87.4)		0.389	0.575
**Total**	222 (100)	46 (100)	0.389	0.575

### Relationship between the employees’ demographics factors and SHS exposure in the workplace

A multiple linear regression analysis was performed to explore the relationship between the employees’ demographic factors and the airborne nicotine and salivary cotinine concentrations (Table 2). The results show that the educational status of employees, average number of smokers per day in the workplace, and exposure time to SHS in the workplace were significantly related to airborne nicotine concentration. According to these observations, a higher employee educational status is associated with lower air nicotine concentration in the workplace (β = –0.286; 95% confidence interval (CI): –0.663, –0.105); the greater the number of smokers per day in the workplace, the higher the air nicotine concentration (β = 0.141; 95% CI:0.003, 0.401). A longer exposure time to SHS was associated with higher airborne nicotine concentration (β = 0.193; 95%CI: 0.143, 0.918). Concerning salivary cotinine concentration, age and educational status of employees as well as exposure time to SHS in the workplace were significant predictors of salivary cotinine concentration. Employees aged 36–45 years showed a β of -0.153 (95% CI: –0.408, –0.034) compared with employees aged 18–35 years. The likelihood of SHS exposure decreased with increasing age. Consistent with the results of nicotine concentrations in the workplace, a higher educational status was associated with a lower salivary cotinine concentration among employees (β = –0.241; 95% CI: –0.637, –0.052). Employee salivary cotinine concentration is positively correlated with SHS exposure time in the workplace (β = 0.257; 95% CI: 0.184, 0.631; β = 0.217; 95% CI: 0.229, 1.042).

**Table 2:** Multivariate linear regression analysis of the employees’ demographics factors associated with airborne nicotine concentration and salivary cotinine concentration (n = 222).

Variable	Airborne nicotine					Salivary cotinine				
	β	95% CI		t	p	Β	95% CI		t	p
**Intercept**		–1.091	–0.273	–3.285	0.001		–0.610	0.249	–0.828	0.409
**Sex**										
**Male**	Referent									
**Female**	0.120	–0.009	0.345	1.867	0.063	–0.097	–0.330	0.042	–1.529	0.128
**Age**										
**18–30**	Referent									
**31–45**	–0.039	–0.232	0.125	–0.593	0.554	–0.153	–0.408	–0.034	–2.331	0.021
**46–60**	–0.070	–0.648	0.193	–1.066	0.288	0.030	–0.339	0.543	0.455	0.650
**Educational status**										
**Low**	Referent									
**Middle**	–0.071	–0.388	0.188	–0.684	0.495	–0.087	–0.432	0.172	–0.849	0.397
**High**	–0.286	–0.663	–0.105	–2.714	0.007	–0.241	–0.637	–0.052	–2.319	0.021
**Average number of smokers per day in the workplace**										
**<1**	Referent									
**1~10**	0.141	0.003	0.401	2.001	0.047	0.080	–0.087	0.330	1.149	0.252
**>10**	0.087	–0.136	0.544	1.182	0.239	–0.035	–0.445	0.269	–0.486	0.628
**Exposure time of SHS in the workplace**										
**<1 h**	Referent									
**1~6h**	0.129	–0.020	0.406	1.784	0.076	0.257	0.184	0.631	3.593	0.000
**>6h**	0.193	0.143	0.918	2.698	0.008	0.217	0.229	1.042	3.081	0.002
**Colleague smoking**										
**Yes**	Referent									
**No**	0.122	–0.019	0.509	1.833	0.068	0.085	–0.095	0.459	1.298	0.196

### Relationship between smoke-free measures and SHS exposure in the workplace

After controlling for employees’ demographic factors, such as gender and age, the relationship between smoke-free measures in workplaces and airborne nicotine and saliva cotinine concentration is shown in Table 3. The division of smoking and non-smoking areas in the workplace was a significant predictor of airborne nicotine concentration. Compared to the workplaces with smoking and non-smoking divisions, the airborne nicotine concentration was higher in workplaces without division (β = 0.193; 95% CI:0.079, 0.482). Tobacco control training and publicity in the workplace were significant predictors of salivary cotinine concentration (p = 0.045). Compared with employees who received tobacco control training and publicity in the workplace, employees who did not receive training had higher airborne nicotine concentrations in the workplace. (β = 0.179; 95% CI:0.006, 0.566).

**Table 3:** Multivariate linear regression analysis of smoke-free measures in workplaces associated with airborne nicotine and salivary cotinine concentration (N = 222).

Variable	Airborne nicotine					Salivary cotinine				
	β	95% CI		t	p	β	95% CI		t	p
**Intercept**		–1.091	–0.273	–3.285	0.001		–0.610	0.249	–0.828	0.409
**Indoor smoking bans**										
**Yes**	Referent									
**No**	0.117	–0.048	0.457	1.596	0.112	0.084	–0.113	0.425	1.146	0.253
**Whether to divide smoking and non-smoking areas**										
**Yes**	Referent									
**No**	0.193	0.079	0.482	2.746	0.007	0.125	–0.020	0.408	1.787	0.075
**Regulations on tobacco control**										
**Yes**	Referent									
**No**	0.027	–0.235	0.33	0.333	0.739	0.071	–0.169	0.432	0.865	0.388
**Leaders in charge of tobacco control**										
**Yes**	Referent									
**No**	0.103	–0.218	0.518	0.804	0.423	–0.101	–0.547	0.236	–0.782	0.435
**Tobacco control supervisor**										
**Yes**	Referent									
**No**	–0.150	–0.587	0.155	–1.147	0.253	–0.087	–0.527	0.261	–0.665	0.507
**Tobacco control training and publicity**										
**Yes**	Referent									
**No**	0.088	–0.131	0.396	0.994	0.321	0.179	0.006	0.566	2.016	0.045
**Employees discourage smoking actively**										
**Yes**	Referent									
**No**	–0.107	–0.731	0.095	–1.518	0.131	–0.112	–0.793	0.086	–1.587	0.114

### Relationship between employer’s tobacco hazard knowledge and attitudes towards smoke-free policy and employee exposure to SHS in the workplace

After controlling for employers’ demographic factors, such as gender and age, the relationship between employers’ tobacco hazard knowledge and attitudes towards smoke-free policy and airborne nicotine and saliva cotinine concentration is shown in Table 4. Employer support of the “Qingdao Tobacco Control Regulation” in the workplace is significantly related to employees’ salivary cotinine concentration. Compared with employers supporting the “Qingdao Tobacco Control Regulation”, employees in workplaces where employers do not support the “Qingdao Tobacco Control Regulation” have higher salivary cotinine concentrations (β = 0.359; 95% CI: 0.050, 1.843).

**Table 4:** Multivariate linear regression analysis of employers’ knowledge and attitudes towards smoking bans associated with airborne nicotine concentration and salivary cotinine concentration (N = 46).

	Airborne nicotine					Salivary cotinine				
	β	95.0%CI		t	P	β	95.0%CI		t	p
**Intercept**		–3.192	0.924	–1.117	0.271		–2.535	0.595	–1.256	0.217
**Whether to support the "Qingdao Tobacco Control Regulation"**										
**Yes**	Referent					Referent				
**No**	0.093	–0.860	1.498	0.548	0.587	0.339	0.050	1.843	2.139	0.039
**The effect of the "Qingdao Tobacco Control Regulation" on the workplace**										
**Beneficial**	Referent					Referent				
**Unhelpful**	–0.138	–0.979	0.407	–0.836	0.408	–0.084	–0.669	0.385	–0.546	0.588
**No effect**	–0.079	–0.980	0.589	–0.504	0.617	–0.180	–0.961	0.233	–1.236	0.224
**Awareness of the "Qingdao Tobacco Control Regulation"**										
**Yes**	Referent					Referent				
**No**	–0.322	–1.492	0.048	–1.901	0.065	–0.037	–0.653	0.518	–0.234	0.816
**Awareness of the maximum fines of the "Qingdao Tobacco Control Regulations"**										
**Yes**	Referent					Referent				
**No**	0.121	–0.333	0.708	0.730	0.470	0.283	–0.038	0.753	1.830	0.075
**Tobacco Hazard Knowledge Score**	0.032	–0.128	0.156	0.198	0.844	0.129	–0.063	0.153	0.847	0.403

## Discussion

Airborne nicotine has been widely used as an indicator of SHS levels in occupational and non-occupational settings [13]. Measurements of airborne nicotine, a tobacco-specific chemical, reflect exposure to tobacco smoke. A passive nicotine sampler is used to measure nicotine in the air with high sensitivity and specificity and has been gradually applied in the evaluation of environmental tobacco smoke pollution in recent years [17]. In this study, we found that the median concentration of airborne nicotine was 0.389 μg/m^3^, which is lower than the partial monitoring results of indoor airborne nicotine in some workplaces in Qingdao in 2016 [17]. However, the workplace remains an environment where further improvements can be made to reduce SHS exposure.

Regarding employee demographics, factors related to airborne nicotine concentrations include educational status and the average number of smokers per day in the workplace. The higher airborne nicotine concentration in the workplace with a lower level of education observed in this study is comparable with previous studies [18, 19]. Those with lower educational attainment and socioeconomic status are less likely to be covered by smoke-free laws in office buildings, restaurants, and bars and are more likely to be exposed to SHS [6]. Additionally, less-educated people have lower awareness of the health effects of smoking [20]. Thus, the development and implementation of evidence-based interventions and tailored strategies are warranted to reduce the exposure rate of SHS among employees in the workplace, with priority given to low educational status groups. The higher the number of smokers per day in the workplace, the greater the airborne nicotine concentration. Other studies have shown that nicotine concentrations increase with the number of cigarettes lit [21, 22], which is consistent with the results of this study. Analysis of fine particulate matter that is 2.5 microns or less in diameter (PM2.5) levels in smoking locations showed an increase of 129 μg/m^3^ in PM2.5 levels per smoker per 100 m^3^ room volume [23]. The PM2.5 concentration is positively correlated with the airborne nicotine concentration [24]; thus, the airborne nicotine concentration increases accordingly. SHS increases exposure to airborne nicotine directly, and when exposed to SHS, nonsmokers inhale 60–80% of airborne nicotine, absorbing concentrations similar to those absorbed by smokers and showing high levels of nicotine biomarkers [25].

Regarding smoke-free measures in the workplace, the division of smoking and non-smoking areas is an important factor influencing the airborne nicotine concentration. The results of this study showed that workplaces with stipulated smoking and non-smoking areas tended to have lower airborne nicotine concentrations. However, a previous study showed that the geometric mean PM2.5 levels in non-smoking rooms are much higher than in completely smoke-free reception venues, even if the non-smoking and smoking areas were spatially separated into two rooms [23]. As early as 2006, a US Surgeon General report concluded that scientific evidence consistently showed that mechanical systems and separate areas could not protect the population from SHS exposure [26]. This study only investigated whether the workplace was divided into smoking and non-smoking areas. Workplaces that are not divided may include completely smoke-free places and places that are not smoke-free, causing certain deviation in the results by bringing in a lot of variance.

Cotinine is the main metabolite of nicotine, and its concentration in body fluids is determined by nicotine metabolism rate and cotinine clearance. Although there may be individual differences in salivary cotinine concentrations due to these parameters, it remains an important indicator of nicotine dependence [27]. The results showed that the median salivary cotinine concentrations of employees in restaurants, bars, and office buildings were 1.165, 0.395, and 0.210 ng/mL, respectively. Restaurants and bars are the greatest sources of SHS exposure for more than half of the non-smoking adults. For non-smoking servers living in smoke-free homes, the time spent in restaurants and bars still dominates the total SHS exposure time [28]. Exposure to SHS in restaurants and bars alone poses a much higher than acceptable health risk of developing asthma among customers and servers, as well as death from cancer and heart disease [29]. The results of this study suggest that age is a significant predictor of salivary cotinine levels, with younger employees having higher salivary cotinine levels. A study from Germany [19] highlighted higher SHS exposure among young people, which is consistent with the results of this study. The educational status and exposure time to SHS are important factors affecting salivary cotinine levels among employees, which is consistent with the analyses of airborne nicotine concentrations in the workplace.

Tobacco control training, awareness of employees, and employer support for the “Qingdao Tobacco Control Regulation” in the workplace were also significant predictors of employees’ salivary cotinine levels. More training for the employees may increase their knowledge about smoking hazards and improve their support for tobacco control in the workplace [30], thereby promoting the implementation of smoke-free policies in the workplace. Employers are the most immediate authority in the workplace [31]. Employers should support the “Qingdao Tobacco Control Regulation” and should be more inclined to devise plans to increase knowledge and attitude towards tobacco control of employees through training programs and awareness campaigns [10]; and reducing employee exposure to SHS in the workplace, thereby reducing employees’ cotinine levels. In contrast, employers with poor attitudes toward smoke-free policies may result in poor actions towards preventing SHS exposure.

The strong positive association between airborne nicotine and salivary cotinine validates the use of either measure as an index of employee SHS exposure in the workplace. Our findings suggest that smoke-free measures and employers’ attitudes toward tobacco control are important in reducing SHS exposure in the workplace. Reducing SHS exposure in the workplace requires joint efforts from employers and employees.

### Limitations and strengths

This study had some limitations that require acknowledgment. Our analysis was limited to non-smoking employees, even though smoking employees experience negative health effects from SHS, in addition to the effects of active smoking. Second, we included exposure that only occurred in the workplace, although people may also be exposed to SHS in other settings such as homes, parks, public buildings, and other venues. Moreover, the small sample size is also a limitation of this study, and while comparable to previously published research, it cannot be adjusted for SHS exposure by the type of business/work. Future research should further expand the population and scope of SHS exposure, and comprehensively evaluate the negative effects of SHS exposure.

This study had several strengths. Our measures of SHS exposure have advantages over those used in other studies. An important strength of this study is the assessment of SHS exposure by measuring airborne nicotine concentrations, a specific tracer often used as a surrogate for other toxic and carcinogenic components in tobacco [32]. Measuring airborne nicotine concentrations allowed us to precisely quantify SHS exposure levels and compare them with previously reported measurements in other countries [22, 33]. The use of salivary cotinine levels as a specific biomarker of SHS exposure in the past 2–5 days is another strength of this study, in addition to the analytical method used for evaluating the salivary cotinine levels being highly sensitive.

## Conclusion

Despite the implementation of the “Qingdao Smoking Control Regulations” in 2013, the workplace remains an important site for SHS exposure. The ‘SHS issue’ has not yet been ‘solved’ and the public health community needs to continue their efforts and consider taking further measures to protect non-smokers from SHS. Therefore, not only should legislation be implemented, public health strategies must also be considered, such as raising workers’ awareness of the risks associated with SHS exposure through health education and developing different smoking prevention and cessation programs to promote a total non-smoking workplace.

## Supporting information

S1 Dataset (XLSX)

S2 Dataset (XLSX)
